# Subtype-specific effects of clonal hematopoiesis on cerebrovascular and cardiometabolic disease risk

**DOI:** 10.3389/fneur.2026.1830391

**Published:** 2026-04-28

**Authors:** Wenqiang Zhu, Miao Tian, Zihan Zhao, Xiaoquan Rao

**Affiliations:** 1 Department of Internal Medicine, Division of Cardiology, Tongji Hospital, Tongji Medical College, Huazhong University of Science and Technology, Wuhan, Hubei, China; 2 Interventional Center of Valvular Heart Disease, Beijing Anzhen Hospital, Capital Medical University, Beijing, China

**Keywords:** cardiometabolic risk, cerebrovascular disease, clonal hematopoiesis of indeterminate potential (chip), Mendelian randomization, stroke

## Abstract

**Background:**

Cerebrovascular and related cardiometabolic diseases frequently cluster in aging populations characterized by metabolic dysfunction and chronic inflammation. Clonal hematopoiesis of indeterminate potential (CHIP) has emerged as an age-related modifier associated with inflammation, metabolic disturbance, and vascular risk. However, whether CHIP exerts subtype-specific effects on cerebrovascular outcomes within a broader cardiometabolic context remains incompletely understood.

**Objectives:**

This study aimed to investigate the causal associations of CHIP and its major genetic subtypes with cerebrovascular and cardiovascular diseases, while examining cancer outcomes as broader systemic context.

**Methods:**

We performed Mendelian randomization analyses of overall CHIP and its five subtypes (DNMT3A, TET2, JAK2, TP53, and ASXL1) in relation to 20 cerebrovascular and cardiovascular diseases and 19 site-specific cancers. Complementary in vitro experiments were conducted to validate the biological contribution of the key subtype under inflammatory and metabolic stress conditions.

**Results:**

Genetically predicted CHIP showed marked heterogeneity across cerebrovascular and cardiovascular outcomes. Among the CHIP subtypes, TET2-CHIP showed the clearest cerebrovascular signal, with significant associations with ischemic stroke, intracerebral hemorrhage, and hypertension. ASXL1-CHIP also showed directional risk elevations for intracerebral hemorrhage and hypertension. In contrast, JAK2-CHIP exhibited inverse associations with intracerebral hemorrhage and atrial fibrillation, whereas DNMT3A-CHIP was positively associated with atrial fibrillation and inversely associated with abdominal aortic aneurysm. Cancer analyses showed additional subtype-specific associations across disease outcomes. Experimental studies further showed that *TET2* deficiency promoted macrophage lipid accumulation and inflammatory activation and induced endothelial dysfunction, supporting the biological relevance of the cerebrovascular and cardiometabolic associations.

**Conclusions:**

CHIP is associated with subtype-specific patterns of cerebrovascular risk within a broader cardiometabolic context. TET2-CHIP showed the most consistent associations with cerebrovascular outcomes. The marked heterogeneity across CHIP subtypes indicates that clonal hematopoiesis should not be considered a uniform exposure, but rather a mutation-defined condition with distinct clinical consequences.

## Introduction

Cerebrovascular diseases frequently arise in the context of metabolic syndrome and chronic inflammation, yet the upstream biological processes linking cardiometabolic risk to stroke vulnerability remain incompletely defined (1, 2). Clonal hematopoiesis of indeterminate potential (CHIP), an age-related expansion of hematopoietic clones carrying somatic driver mutations, has emerged as a potential systemic inflammatory modifier associated with metabolic dysfunction and vascular disease (3–8). Whether CHIP contributes causally to cerebrovascular risk within this broader cardiometabolic framework, however, remains uncertain.

Growing evidence from observational studies has linked CHIP to cardiovascular diseases, including myocardial infarction (9), heart failure (10), atrial fibrillation (11, 12), and stroke (13). However, these epidemiological findings are constrained by inherent limitations of cohort designs, such as residual confounding from shared risk factors (e.g., aging, smoking, and metabolic comorbidities), resulting in inconsistent associations across studies and complicating causal inference. Meanwhile, experimental and mechanistic studies support a pro-inflammatory role of CHIP in vascular pathology, yet most mechanistic investigations have focused on atherosclerotic cardiovascular disease (14–16), leaving its relevance to cerebrovascular outcomes and broader disease clustering insufficiently characterized.

CHIP is also linked to malignancy. Beyond its established role in hematologic cancers, CHIP has been detected in multiple solid tumors and cancer survivors (3, 17–19). Because cancer frequently coexists with cerebrovascular and cardiovascular disease in aging populations, examining cancer outcomes may help place the vascular findings in a broader systemic context. Examining cancer outcomes alongside cerebrovascular phenotypes therefore provides a framework to determine whether CHIP exerts shared, divergent, or subtype-specific effects across disease systems.

A further limitation of current research lies in the inadequate dissection of CHIP by genetic subtype. CHIP is most commonly driven by mutations in *DNMT3A, TET2, JAK2, TP53*, and *ASXL1*, and its prevalence increases substantially with age (3). Distinct driver mutations may differentially influence inflammatory signaling, metabolic regulation, and vascular biology, but experimental and clinical studies have often focused on selected mutations or specific disease contexts. Clarifying such subtype-specific patterns is essential for refining cerebrovascular and cardiometabolic risk stratification.

To address these gaps, we performed a Mendelian randomization study to evaluate the causal associations of genetically predicted CHIP and its major subtypes with cerebrovascular and cardiovascular diseases, while also examining site-specific cancers as broader systemic context. In addition, complementary experimental validation was performed using macrophages and endothelial cells, considering that CHIP arises from hematopoietic clones and may influence vascular pathology through interactions between myeloid cells and the vascular endothelium. By elucidating subtype-specific pathogenic effects and integrating genetic evidence with cellular validation, our study aims to provide new insights into the systemic and vascular consequences of CHIP and inform future precision risk assessment strategies.

## Methods

### Study design

**Figure 1:**
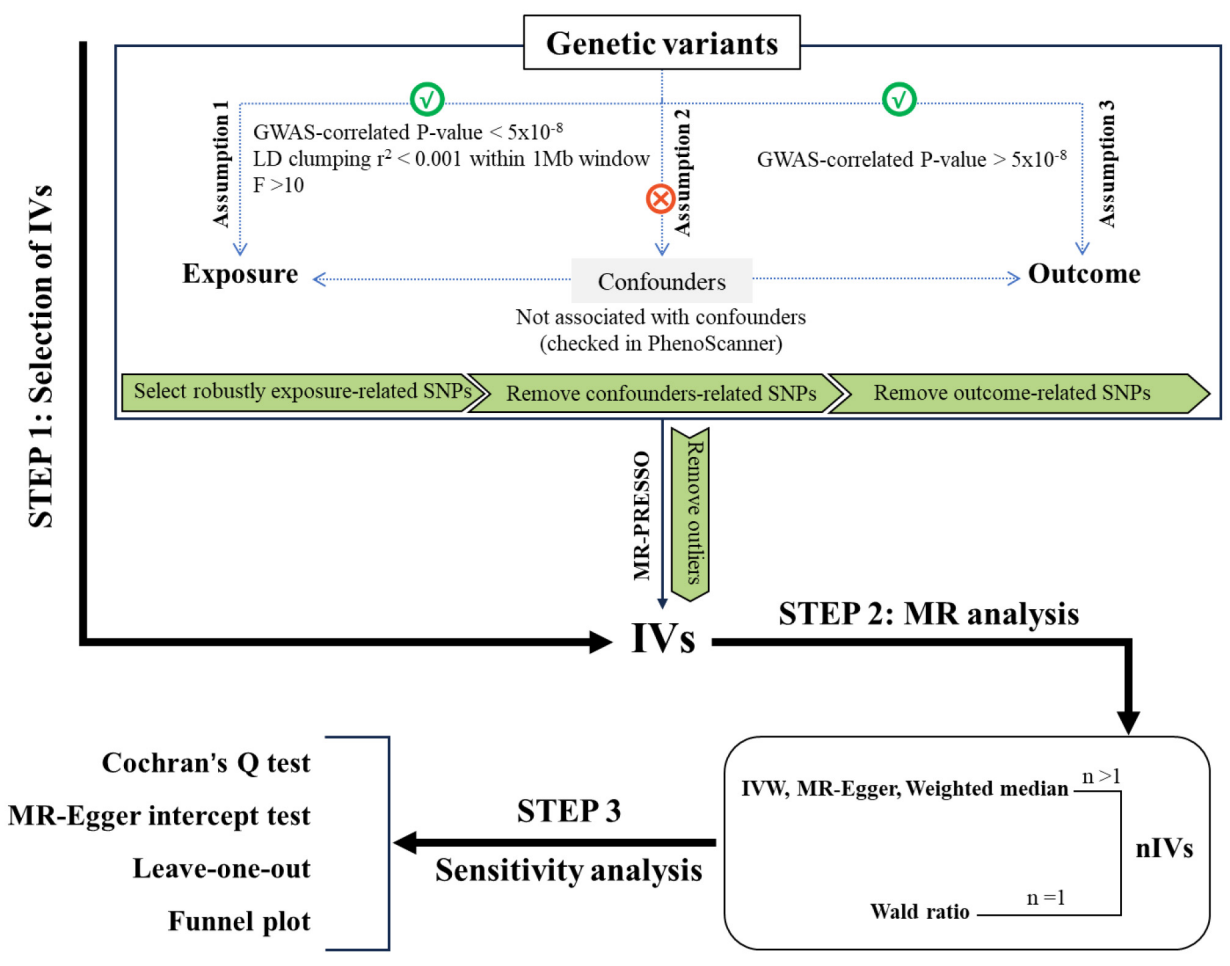
Study design and flowchart of MR analysis in this study.

MR analysis was utilized to investigate the causal relationships of CHIP and its subtypes (DNMT3A-CHIP, TET2-CHIP, JAK2-CHIP, TP53-CHIP and ASXL1-CHIP) on 19 site-specific cancers and 20 cerebrovascular and cardiovascular diseases. The study design is shown in Figure 1. The MR analysis was conducted using genome-wide association study (GWAS) summary statistics and was based on three core assumptions: (1) Genetic instruments exhibit strong associations with exposures; (2) Genetic instruments are independent of confounding factors; (3) Genetic instruments affect the outcome solely via the exposures of interest (20).

### Data source

Single-nucleotide polymorphisms (SNPs) associated with CHIP and CHIP subtypes were obtained from a recent large-scale genome-wide association study involving 368,526 individuals of European ancestry. This study represents the largest assessment to date of individuals with CHIP mutation carrier information and identified novel common and rare variant loci associated with CHIP and its subtypes (21). Cardiovascular diseases and site-specific cancers data were sourced from the FinnGen consortium's R9 release, accessible to the public on their website: https://r9.risteys.finngen.fi/. Detailed information on disease definitions, controls, and supplementary MR results is provided in Supplementary Tables S1–S13.

### Instrumental variable (IV) selection

IV selection adhered to the following stringent criteria: 1) extraction of SNPs at genome-wide significance (*P* < 5 × 10^−8^), 2) removal of SNPs exhibiting linkage disequilibrium (LD, *r*^2^ ≥ 0.001, clumping distance > 10,000 kb) for independence, 3) exclusion of SNPs with harmonization issues, including palindromic, duplicate and incompatible SNPs, 4) Assessment of SNPs via PhenoScanner [Accession at: http://www.phenoscanner.medschl.cam.ac.uk/; (37)] to avoid pleiotropy and 5) *F* statistics were calculated to exclude weak instruments (*F* < 10) using the following formula: $F=\frac{R^{2}\;\times \;\left(N-1-K\right)}{\left(1-R^{2}\right)\;\times \;K}$, [*R*^2^=2 × β^2^ × eaf × (1–eaf), eaf = effect allele frequency, *N* = sample size, *K* = number of IVs]. Finally, Mendelian Randomization Pleiotropy Residual Sum and Outlier (MR-PRESSO) tests were employed to identify and address horizontal pleiotropy by removing outliers.

### Mendelian randomization analyses

The analytical strategy involved differentiating between CHIP and CHIP subtypes based on IV availability: the Wald ratio was used for single IV, and the inverse-variance weighted (IVW) method for multiple IVs. The primary causal estimates were obtained using the IVW method, which employs a multiplicative random-effects model to combine the Wald ratio of each SNP on the outcome, yielding a pooled causal estimate (22). This method generally provides the greatest statistical power when all IVs are valid, although it may be susceptible to pleiotropic bias. Complementary methods, including weighted median and MR-Egger regression, were utilized alongside IVW (23). The weighted median approach yields consistent estimates if at least half of the weighted variance is valid. MR-Egger regression can produce consistent estimates by adjusting for pleiotropy, even when all IVs are invalid, but it may suffer from low power. When the results of the three MR methods are inconsistent, causal inference primarily relies on the IVW method.

### Sensitivity analysis

Comprehensive sensitivity analyses included MR-Egger intercept for horizontal pleiotropy assessment, Cochran's Q test and funnel plots for heterogeneity and influence evaluation (23). A leave-one-out (LOO) analysis was conducted to assess whether the pooled estimation was affected by any influential data points. These analyses were performed to assess the robustness of the findings and to reduce the influence of individual variants or horizontal pleiotropy.

### Cell culture

THP-1 cells were maintained in RPMI-1640 medium supplemented with 10% fetal bovine serum (FBS) and 1% penicillin-streptomycin. Human umbilical vein endothelial cells (HUVECs) were cultured in Dulbecco's modified Eagle's medium (DMEM) supplemented with 10% FBS and 1% penicillin-streptomycin. All cells were maintained at 37 °C in a humidified incubator with 5% CO_2_.

To obtain macrophage-like cells, THP-1 cells were differentiated by treatment with phorbol 12-myristate 13-acetate (HY-18739, MCE, USA) at a concentration of 100 nm for 48 h. After differentiation, cells were washed and cultured in fresh medium prior to subsequent experiments. HUVECs were passaged 2–3 times per week and used within 30 passages after thawing.

### siRNA transfection

For *TET2* knockdown, THP-1-derived macrophages were transfected with small interfering RNA targeting human *TET2* (si-*TET2*) or a non-targeting negative control siRNA (si-NC) using Lipofectamine™ 2000 Transfection Reagent (11668019, Thermo Scientific, USA) according to the manufacturer's instructions. The siRNA sequences targeting human *TET2* were obtained from previously published studies (24). The sequences were as follows:

si-*TET2* sense: 5′-ACCUCAGGGCAGAUCAAUU(dT)(dT)-3′

si-*TET2* antisense: 5′-AAUUGAUCUGCCCUGAGGU(dT) (dT)-3′

Cells were transfected with siRNA at a final concentration of 50 nm and incubated for 48 h to achieve gene silencing. The knockdown efficiency of *TET2* was verified by quantitative real-time PCR.

### Cell treatments

After transfection, cells were subjected to inflammatory or metabolic stimulation. For inflammatory stimulation, cells were treated with lipopolysaccharide (LPS; HY-D1056, MCE, USA) at a final concentration of 100 ng/mL. For metabolic stress stimulation, cells were treated with oxidized low-density lipoprotein (oxLDL; YB-002, Yiyuan Biotechnologies, China) at a final concentration of 50 μg/mL. Cells were divided into four groups: si-NC, si-*TET2*, LPS/oxLDL + si-NC, and LPS/oxLDL + si-*TET2*. After stimulation for 24 h, culture supernatants were collected and centrifuged to remove cell debris. The conditioned media were then applied to HUVECs for 24 h before downstream analyses.

### BODIPY staining

Intracellular lipid accumulation in THP-1-derived macrophages was assessed using BODIPY staining to evaluate whether *TET2* knockdown influences macrophage lipid metabolism under metabolic stress conditions. After siRNA transfection and oxLDL stimulation, cells were washed twice with phosphate-buffered saline (PBS) and fixed with 4% paraformaldehyde for 15 min at room temperature. Cells were then incubated with BODIPY 493/503 (D3922, Thermo Fisher Scientific, USA) at a final concentration of 1 μg/mL for 30 min in the dark to visualize neutral lipid droplets. After staining, cells were washed with PBS and nuclei were counterstained with DAPI for 5 min. Fluorescence images were captured using a fluorescence microscope. Lipid accumulation was quantified by measuring BODIPY fluorescence intensity using ImageJ software. All experiments were performed independently at least three times.

### Quantitative real-time PCR

Quantitative real-time PCR (qPCR) was performed as previously described (25). Briefly, total RNA was extracted, reverse-transcribed into cDNA, and analyzed using SYBR Green-based real-time PCR. Relative gene expression was normalized to *GAPDH* and calculated using the 2^−ΔΔ^Ct method. Based on MR findings, inflammatory markers (*TNFA, IL6, IL1B, CCL2*, and *NLRP3*) were examined in THP-1-derived macrophages, while endothelial injury markers (*ICAM1, VCAM1, CDH5*, and *NOS3*) were assessed in HUVECs to evaluate vascular responses. The primer sequences used for qPCR analysis are provided in Supplementary Table S14.

### Statistical analysis

Stringent standards for statistical significance were implemented, adopting the false discovery rate correction for multiple testing when number of IVs > 1. The corrected *q*-value was calculated as (*p* × *m*) / *k*, where *m* denoted the number of MR analysis methods and *k* represented their respective ranking when *p*-values were sorted from smallest to largest. For single SNP estimation, the *q*-value was equal to the *p*-value. The significance threshold was set at *q* < 0.05. A nominally significant estimate was defined as *p* < 0.05 but *q* ≥ 0.05. Analyses were performed using the TwoSampleMR package (version 0.5.7) in *R* (version 4.3.1), ensuring precision and replicability. For experimental validation, data are presented as mean ± standard deviation (SD) from at least three independent experiments. Comparisons between two groups were performed using unpaired Student's t-test, while multiple group comparisons were analyzed using one-way ANOVA followed by Tukey's post hoc test. Statistical analyses were performed using GraphPad Prism (version 8.0.1). A two-tailed *p*-value < 0.05 was considered statistically significant.

## Results

### SNP inclusion

After instrument selection, independent and robust IVs were retained for MR analyses. Specifically, we identified 23 SNPs for CHIP, 19 SNPs for DNMT3A-CHIP, 5 SNPs for TET2-CHIP, 1 SNP for JAK2-CHIP, and 2 SNPs for ASXL1-CHIP at genome-wide significance (*P* < 5 × 10^−8^), respectively. Specifically, for TP53-CHIP, we identified one SNP for the cerebrovascular/cardiovascular disease analyses, whereas no suitable SNP was available for the cancer analyses. The *F* statistics for these SNPs ranged from 505 to 123,858, indicating sufficiently strong instruments. Additionally, any SNPs identified as outliers by MR-PRESSO (global test: *p* < 0.05) were excluded from further analysis. Two SNPs (rs8088824 and rs2396004) that showed associations with blood pressure, and another SNP (rs7705526) that showed associations with chronic lymphocytic leukemia, ovarian cancer, and lung adenocarcinoma, were removed upon examination in PhenoScanner. These steps helped reduce the likelihood of bias arising from pleiotropic instrumental effects. Detailed information regarding the retained SNPs and relevant statistical data for the cerebrovascular and cardiometabolic analyses can be found in Supplementary Table S1, whereas the corresponding information for the cancer analyses is provided in Supplementary Table S8.

### Overview of MR analysis

**Figure 2:**
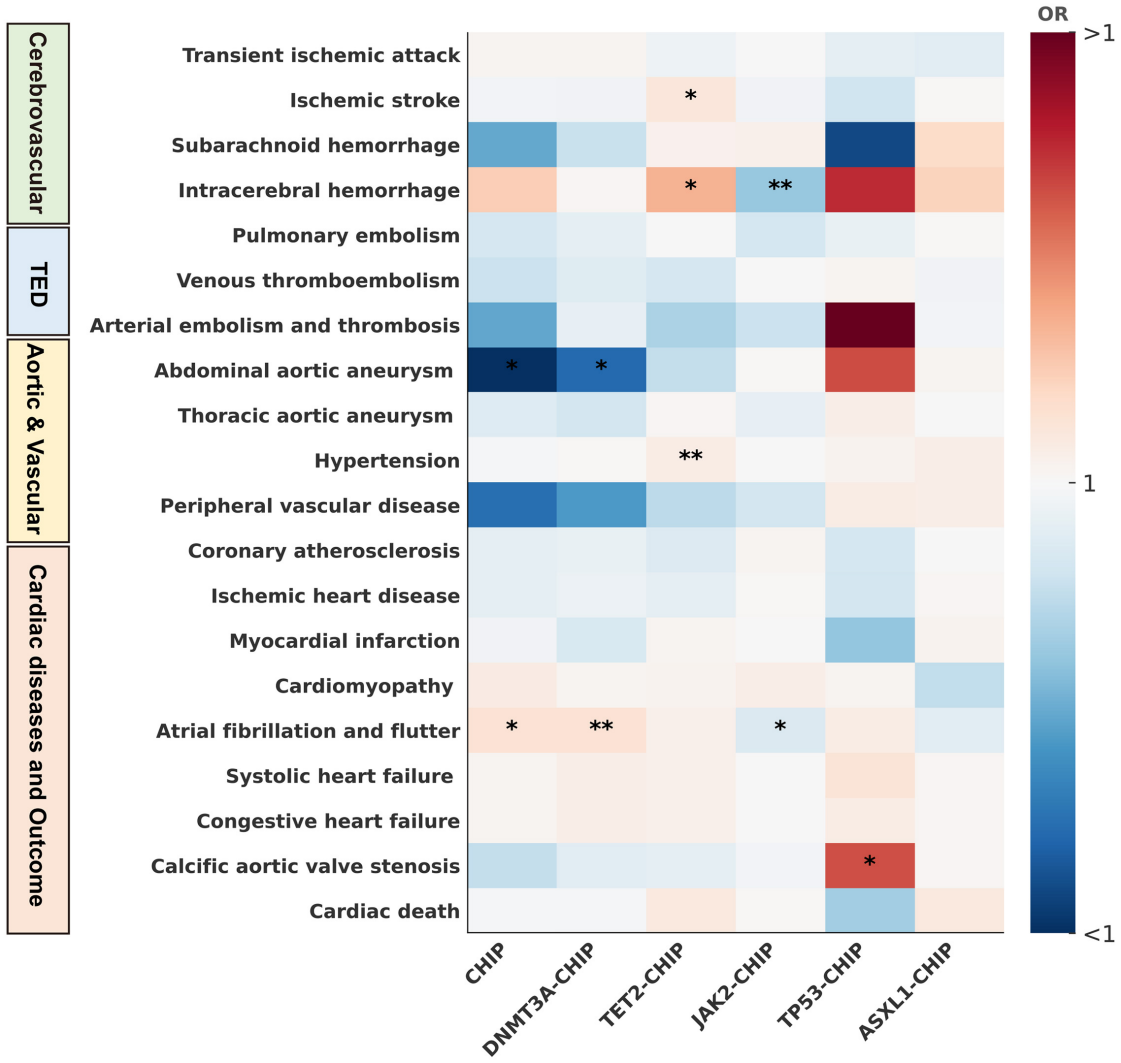
Heatmap of CHIP and its subtypes with 20 cerebrovascular and cardiovascular diseases. This figure illustrates causal associations between CHIP and its subtypes (DNMT3A-CHIP, TET2-CHIP, JAK2-CHIP, TP53-CHIP, ASXL1-CHIP) with 20 CVDs, identified via Mendelian randomization analyses. TED, thromboembolic diseases; ^*^P or *q*-values < 0.05; ^**^P or *q*-values < 0.01.

**Figure 3:**
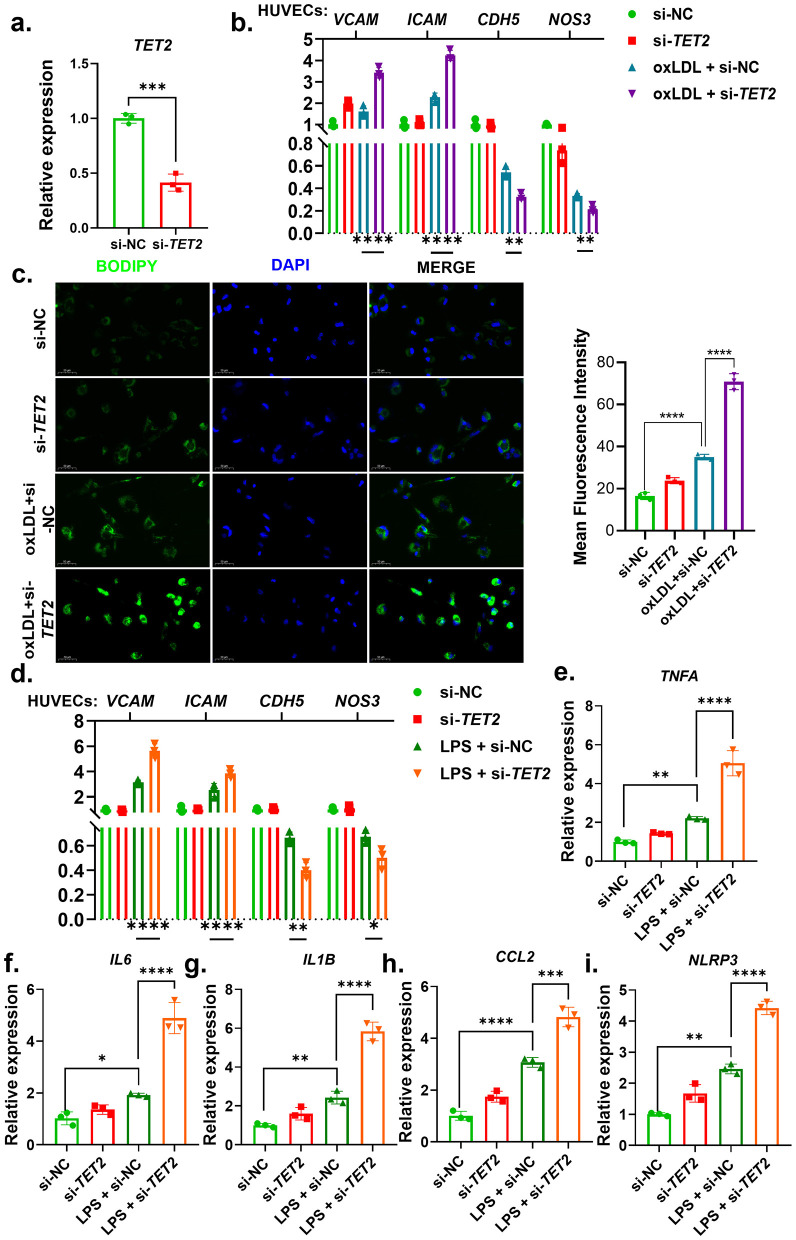
*TET2* knockdown promotes macrophage metabolic and inflammatory activation and aggravates endothelial dysfunction. **(a)** Knockdown efficiency of *TET2* in THP-1-derived macrophages was confirmed by qPCR after siRNA transfection. **(b)** HUVECs were treated with conditioned media from oxLDL-stimulated THP-1-derived macrophages, and the mRNA expression levels of endothelial activation markers (*ICAM1* and *VCAM1*) and endothelial functional markers (*CDH5* and *NOS3*) were measured by qPCR. **(c)** Representative BODIPY staining images and quantification of intracellular lipid accumulation in THP-1-derived macrophages following oxLDL treatment with or without *TET2* knockdown. **(d)** HUVECs were treated with conditioned media from LPS-stimulated THP-1-derived macrophages, and the expression of endothelial activation and functional markers was analyzed by qPCR. **(e–i)** Relative mRNA expression levels of inflammatory genes (*TNFA, IL6, IL1B, CCL2*, and *NLRP3*) in THP-1-derived macrophages following LPS stimulation with or without *TET2* knockdown. ^*^P < 0.05; ^**^P < 0.01; ^***^P < 0.001; ^****^P < 0.0001.

We conducted a comprehensive Mendelian randomization analysis to evaluate the associations of genetically predicted CHIP and its major subtypes with a wide spectrum of cerebrovascular and cardiovascular diseases, as well as 19 cancers, using IVW/Wald ratio, weighted median, and MR-Egger methods, identifying several significant and nominal associations (Tables 1, 2 and Figures 2, 3). The scatter plots of significant and nominally significant estimates are shown in Supplementary Figure S1, S2, S7, S8. For most outcomes, the weighted median and MR-Egger analyses showed estimates in the same general direction as the IVW results, although with lower precision (Supplementary Tables S2–S7, S9–S13).

**Table 1:** Significant and nominally significant estimates of causal associations between chip and cardiovascular and cerebrovascular diseases.

Estimate category	Exposure	Outcome	MR method	*p*	*q*	OR (95% confidence intervals)	Cochran's *Q*-derived *P-*value	MR-Egger intercept-derived *P*-value
Significant estimates^*^	CHIP	Abdominal aortic aneurysm	IVW	**6.30** **×10**^**−4**^	**0.01**	0.80 (0.70–0.91)	0.19	0.93
		Atrial fibrillation and flutter	IVW	**1.06** **×10**^**−3**^	**0.01**	1.09 (1.03–1.14)	0.34	0.28
	DNMT3A-CHIP	Abdominal aortic aneurysm	IVW	**4.87** **×10**^**−3**^	**0.049**	0.84 (0.75–0.95)	0.14	0.32
		Atrial fibrillation and flutter	IVW	**1.21** **×10**^**−4**^	**2.41** **×10**^**−3**^	1.09 (1.04–1.13)	0.79	0.24
	TET2-CHIP	Ischemic stroke	IVW	**4.96** **×10**^**−3**^	**0.03**	1.07 (1.02–1.12)	0.57	0.98
		Intracerebral hemorrhage	IVW	**6.27** **×10**^**−4**^	**0.01**	1.21 (1.09–1.35)	0.51	0.29
		Hypertension	IVW	**7.51** **×10**^**−4**^	**7.51** **×10**^**−3**^	1.05 (1.02–1.08)	0.44	0.28
	JAK2-CHIP	Intracerebral hemorrhage	Wald ratio	**7.71** **×10**^**−3**^	/	0.92 (0.87–0.98)	/	/
		Atrial fibrillation and flutter	Wald ratio	**0.01**	/	0.97 (0.95–0.99)	/	/
	TP53-CHIP	Calcific aortic valve stenosis	Wald ratio	**0.01**	/	1.40 (1.07–1.82)	/	/
Nominal significant estimates^**^	CHIP	Intracerebral hemorrhage	IVW	**0.04**	0.21	1.15 (1.00–1.32)	0.07	0.98
		Peripheral vascular disease	IVW	**0.02**	0.12	0.85 (0.73–0.97)	0.90	0.33
	ASXL1-CHIP	Intracerebral hemorrhage	IVW	**0.04**	0.42	1.14 (1.00–1.29)	/	/
		Hypertension	IVW	**0.01**	0.23	1.04 (1.01–1.07)	/	/

^*^Significant estimate is defined as both *p* and *q* < 0.05.

^**^Nominal significant estimate is defined as *p* < 0.05 but *q* ≥ 0.05.

Cochran's *Q*-derived *P*-value and MR-Egger intercept-derived *P*-value < 0.05 is significant.

IVW, inverse-variance weighted; MR, Mendelian randomization. Bold values indicate statistically significant estimates based on *p* or *q* values.

**Table 2:** Significant and nominally significant estimates of causal associations between CHIP and 19 site-specific cancers.

Estimate category	Exposure	Outcome	MR method	*P*	*q*	OR (95% confidence intervals)	Cochran's *Q*-derived *P*-value	MR-egger intercept-derived *P*-value
Significant estimates^*^	CHIP	Esophageal cancer	IVW	**1.05** **×10**^**−3**^	**5.00** **×10**^**−3**^	1.76(1.26–2.48)	0.08	0.34
		Liver cancer	IVW	**1.79** **×10**^**−4**^	**1.13** **×10**^**−3**^	0.54 (0.39–0.75)	0.59	0.12
		Thyroid cancer	IVW	**1.85** **×10**^**−3**^	**7.01** **×10**^**−3**^	1.36 (1.12–1.65)	0.07	0.20
		Myeloid leukemia	IVW	**5.39** **×10**^**−5**^	**1.02** **×10**^**−3**^	1.91 (1.40–2.62)	0.46	0.68
		Non-melanoma skin cancer	IVW	**8.00** **×10**^**−5**^	**7.60** **×10**^**−4**^	1.12(1.06–1.18)	0.48	0.97
		Melanoma skin cancer	IVW	**0.01**	**0.04**	1.23 (1.05–1.45)	0.20	0.12
		Bladder cancer	IVW	**0.02**	**0.04**	0.81(0.67–0.96)	0.10	0.47
	DNMT3A-CHIP	Esophageal cancer	IVW	**4.39** **×10**^**−4**^	**8.34** **×10**^**−3**^	1.63 (1.24–2.13)	0.23	0.32
		Liver cancer	IVW	**8.69** **×10**^**−4**^	**5.50** **×10**^**−3**^	0.58(0.42–0.80)	0.15	0.08
		Myeloid leukemia	IVW	**6.98** **×10**^**−4**^	**6.63** **×10**^**−3**^	1.67 (1.24–2.25)	0.21	0.63
		Non-melanoma skin cancer	IVW	**1.96** **×10**^**−3**^	**9.33** **×10**^**−3**^	1.10 (1.04–1.18)	0.045	0.64
	JAK2-CHIP	Esophageal cancer	Wald ratio	**0.04**	/	0.85 (0.73–0.99)		/
		Myeloid leukemia	Wald ratio	**0.02**	/	1.18 (1.02–1.35)		/
Nominal significant estimates^**^	DNMT3A-CHIP	Breast cancer	IVW	**0.02**	0.06	1.06 (1.01–1.12)	0.45	0.67
		Lymphoid Leukemia	IVW	**0.049**	0.13	1.20(1.00–1.44)	0.44	0.46
		Kidney cancer	IVW	**0.02**	0.06	1.17(1.03–1.32)	0.49	0.19
	TET2-CHIP	Myeloid leukemia	IVW	**0.04**	0.76	1.50 (1.02–2.22)	0.21	0.75

^*^Significant estimate is defined as both *p* and *q* < 0.05.

^**^Nominal significant estimate is defined as *p* < 0.05 but *q* ≥ 0.05.

Cochran's *Q*-derived *P*-value and MR-Egger intercept-derived *P*-value < 0.05 is significant.

IVW, inverse-variance weighted; MR, Mendelian randomization. Bold values indicate statistically significant estimates based on *p* or *q* values.

### Causal associations with cerebrovascular and cardiovascular diseases

Subtype-specific analyses revealed marked heterogeneity across cerebrovascular and cardiovascular outcomes (Table 1, Figure 2, Supplementary Tables S2–8). Among the CHIP subtypes, TET2-CHIP showed the clearest cerebrovascular signal, with significant positive associations with ischemic stroke (IS) (*OR* = 1.07; 95% CI, 1.02–1.12; *q* = 0.03), intracerebral hemorrhage (ICH) (*OR* = 1.21; 95% CI, 1.09–1.35; *q* = 0.01), and hypertension (*OR* = 1.05; 95% CI, 1.02–1.08; *p* = 7.51 × 10^−3^). ASXL1-CHIP also showed directionally consistent, nominally significant associations with increased risks of ICH and hypertension (ORs: 1.14 and 1.04, respectively). Using the Wald ratio method for MR estimation, JAK2-CHIP showed significant negative associations with ICH and atrial fibrillation/flutter (ORs: 0.92 and 0.97, respectively). These findings suggest that CHIP may influence cerebrovascular risk both directly and through related clinical phenotypes.

DNMT3A-CHIP showed significant associations with related cardiovascular phenotypes, including increased risk of atrial fibrillation/flutter (*OR* = 1.09; 95% CI, 1.04–1.13; *q* = 2.41 × 10^−3^) and decreased risk of abdominal aortic aneurysm (AAA) (*OR* = 0.84; 95% CI, 0.75–0.95; *q* = 0.049). TP53-CHIP was significantly positively associated with calcific aortic valve stenosis (*OR* = 1.40; 95% CI, 1.07–1.82). In the IVW analysis, genetic predisposition to CHIP showed several significant and nominally significant associations across cerebrovascular and cardiovascular diseases. Specifically, there was suggestive evidence of a positive association between CHIP and ICH (*OR* = 1.15; 95% CI, 1.00–1.32; *p* = 0.04). CHIP was also significantly associated with atrial fibrillation and flutter (*OR* = 1.09; 95% CI, 1.03–1.14; *q* = 0.01), while showing a negative association with AAA (*OR* = 0.80; 95% CI, 0.70–0.91; *q* = 0.01). Additionally, nominal evidence suggested a negative association between CHIP and peripheral vascular disease (*OR* = 0.85; 95% CI, 0.73–0.97; *p* = 0.02).

Overall, these results indicate substantial subtype-specific heterogeneity across cerebrovascular and cardiovascular outcomes, with TET2-CHIP showing the most consistent pattern across major cerebrovascular outcomes and related clinical phenotypes.

### TET2-CHIP promotes macrophage metabolic and inflammatory responses and aggravates endothelial dysfunction

Based on the MR findings, TET2 was selected for experimental validation because it showed the clearest and most consistent pattern of associations across major cerebrovascular outcomes (IS, ICH, and hypertension), suggesting a potentially important role in cerebrovascular vulnerability. Considering that CHIP primarily affects myeloid cells and that endothelial dysfunction represents a central feature of cardiometabolic and cerebrovascular diseases, macrophage-endothelial interaction models were used. First, efficient knockdown of *TET2* in THP-1-derived macrophages was confirmed by qPCR (Figure 3a). We then examined whether conditioned media from metabolically stressed macrophages could affect endothelial cells. HUVECs treated with conditioned media from oxLDL-stimulated macrophages showed increased expression of the endothelial activation markers *ICAM1* and *VCAM1*, together with reduced expression of the endothelial functional markers *CDH5* and *NOS3*. These effects were more pronounced when macrophages were subjected to *TET2* knockdown (Figure 3b). Consistent with this metabolic stress response, BODIPY staining showed that oxLDL induced intracellular lipid accumulation in THP-1-derived macrophages, which was further enhanced after *TET2* knockdown (Figure 3c).

We next assessed inflammatory stimulation using LPS. Similar to the oxLDL-conditioned media, conditioned media from LPS-stimulated macrophages promoted endothelial activation and reduced endothelial functional gene expression in HUVECs, with the strongest effects observed in the LPS + si-*TET2* group (Figure 3d). At the macrophage level, *TET2* knockdown significantly increased the expression of inflammatory genes, including *TNFA, IL6, IL1B, CCL2*, and *NLRP3*, particularly under LPS stimulation (Figures 3e–i). Collectively, these findings indicate that *TET2* deficiency enhances macrophage metabolic and inflammatory responses and aggravates endothelial dysfunction, providing cellular support for the subtype-specific associations of TET2-CHIP with cerebrovascular outcomes and related cardiometabolic phenotypes observed in the MR analysis.

### Causal associations with cancers

**Figure 4:**
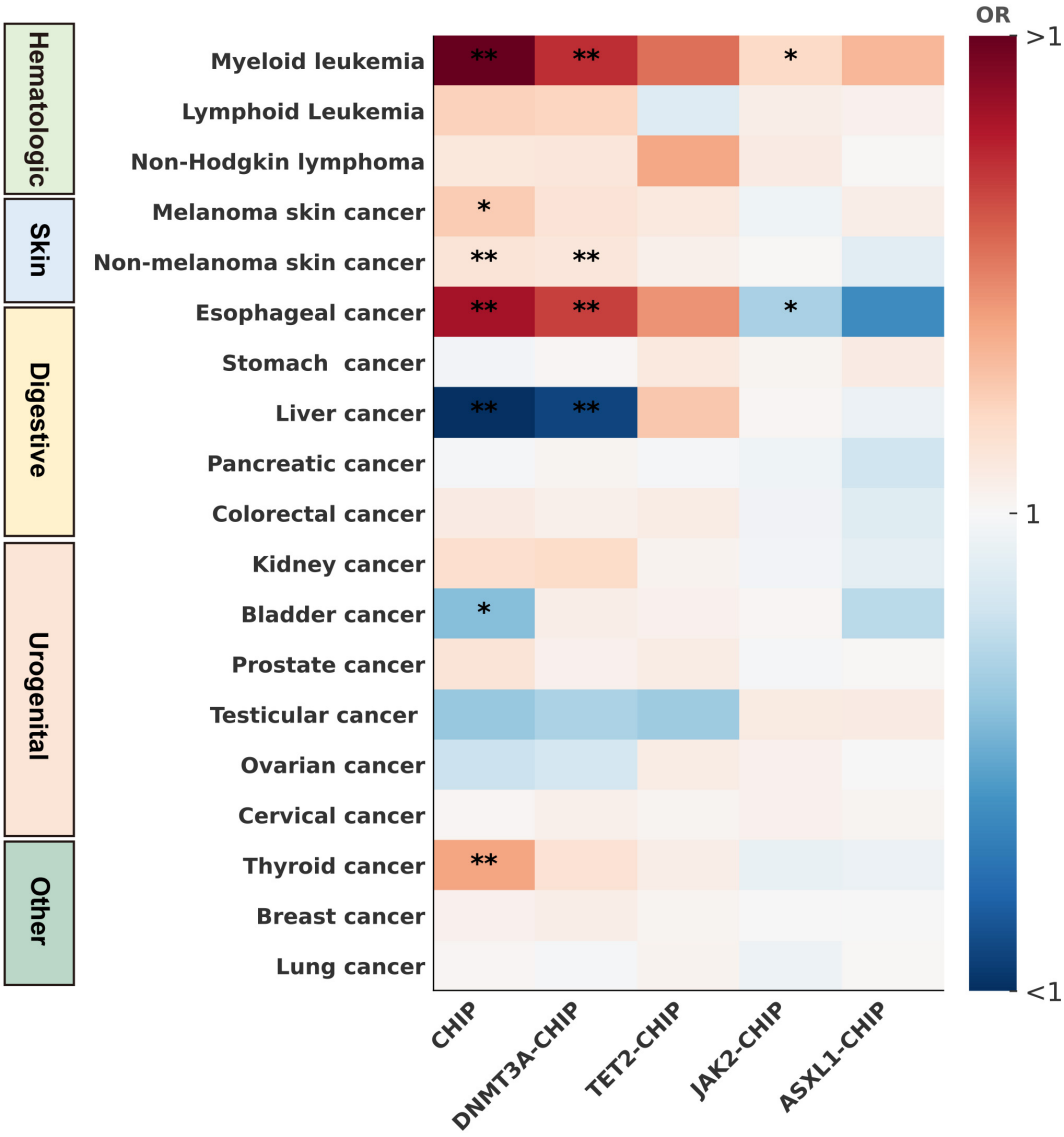
Heatmap of CHIP and its subtypes with19 site-specific cancers. This figure illustrated causal associations between CHIP and its subtypes (DNMT3A-CHIP, TET2-CHIP, JAK2-CHIP, ASXL1-CHIP) with 19 site-specific cancers, identified via Mendelian randomization analyses. ^*^P or *q*-values < 0.05; ^**^P or *q*-values < 0.01.

Cancer outcomes were further examined to place the cerebrovascular and cardiovascular findings in a broader systemic context (Table 2, Figure 4, Supplementary Tables S9–S13). Overall, CHIP was significantly associated with 7 cancer types. Positive associations were observed with esophageal cancer (*OR* = 1.76; 95% CI, 1.26–2.48; *q* = 5 × 10^−3^), thyroid cancer (*OR* = 1.36; 95% CI, 1.12–1.65; *q* = 1.13 × 10^−3^), myeloid leukemia (*OR* = 1.91; 95% CI, 1.40–2.62; *q* = 1.02 × 10^−3^), non-melanoma skin cancer (*OR* = 1.12; 95% CI, 1.06–1.18; *q* = 7.60 × 10^−4^), and melanoma (*OR* = 1.23; 95% CI, 1.05–1.45; *q* = 0.04). In contrast, CHIP showed negative associations with liver cancer (*OR* = 0.54; 95% CI, 0.39–0.75; *q* = 1.13 × 10^−3^) and bladder cancer (*OR* = 0.81; 95% CI, 0.67–0.96; *q* = 0.04). No statistically significant associations were observed for the remaining 12 cancers.

Subtype-specific analyses also revealed distinct patterns across cancers. DNMT3A-CHIP significantly increased the risk of esophageal cancer (*OR* = 1.63; 95% CI, 1.24–2.13; *q* = 8.34 × 10^−3^), myeloid leukemia (*OR* = 1.67; 95% CI, 1.24–2.25; *q* = 6.63 × 10^−3^), and non-melanoma skin cancer (*OR* = 1.10; 95% CI, 1.04–1.18; *q* = 9.33 × 10^−3^), while decreasing liver cancer risk (*OR* = 0.58; 95% CI, 0.42–0.80; *q* = 5.50 × 10^−3^). Suggestive associations indicated increased risks of breast cancer, lymphoid leukemia, and kidney cancer. TET2-CHIP showed suggestive positive associations with myeloid leukemia (*OR* = 1.50; 95% CI, 1.02–2.22; *p* = 0.04). JAK2-CHIP was associated with decreased risk of esophageal cancer (*OR* = 0.85; 95% CI, 0.73–0.99; *p* = 0.04) and increased risk of myeloid leukemia (*OR* = 1.18; 95% CI, 1.02–1.35; *p* = 0.02). ASXL1-CHIP showed no significant associations with any cancer outcomes.

### Sensitivity analyses

Cochran's *Q* statistic (Tables 1, 2; all *P*-values > 0.05 except for non-melanoma skin cancer, which had a *P*-value of 0.045) and the funnel plot (Supplementary Figures S3, S4, S9, S10) indicated no evidence of heterogeneity. The MR-Egger intercept test (Tables 1, 2; all *P*-values > 0.05) did not suggest potential horizontal pleiotropy. Furthermore, our findings remained stable when re-analyzing the data after removing one SNP each time during LOO test (Supplementary Figures S5, S6, S11, S12).

## Discussion

**Figure 5:**
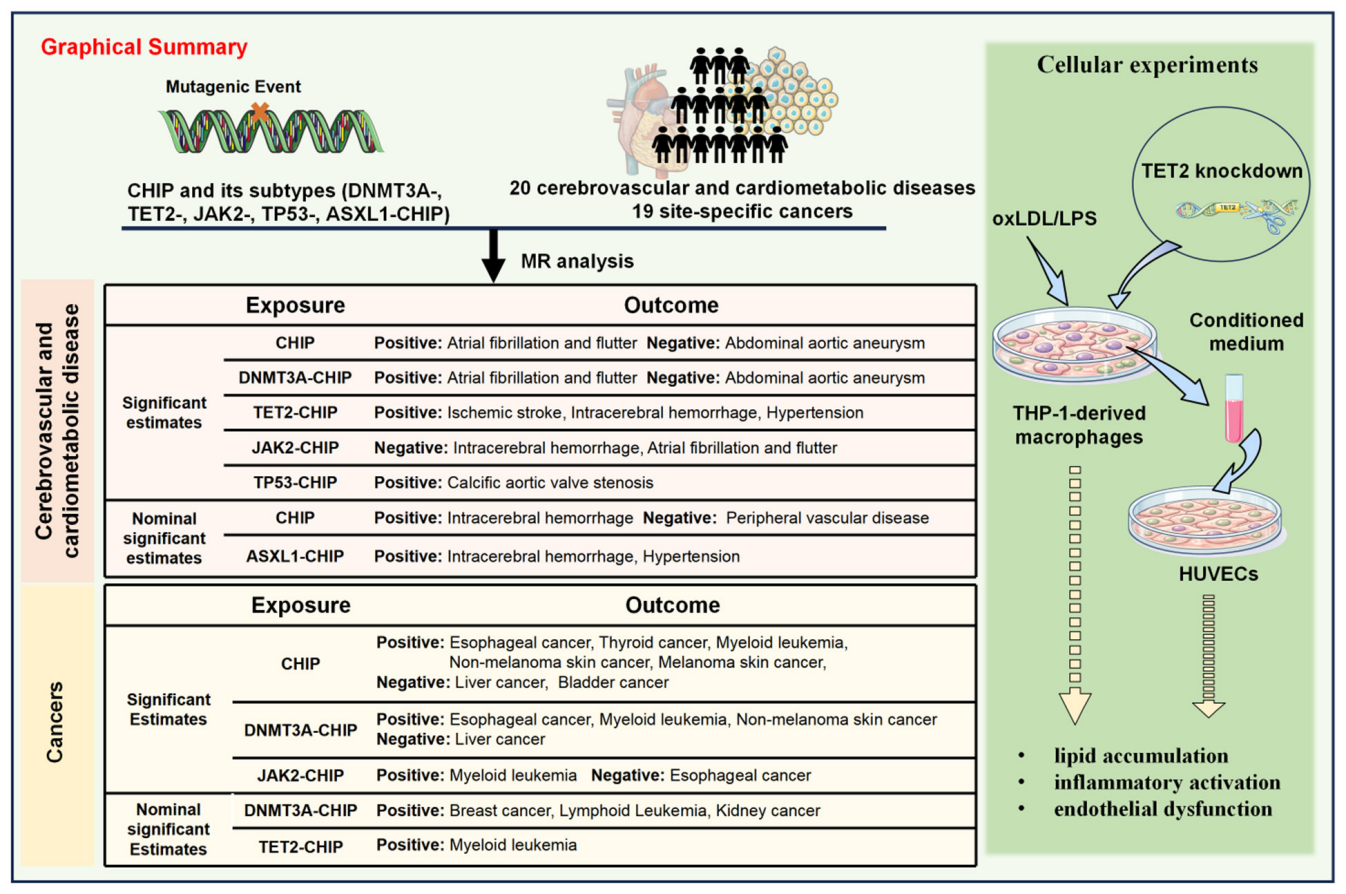
Graphical summary the main subtype-specific associations of CHIP across cerebrovascular and cardiovascular outcomes, cancer outcomes, and supporting experimental evidence. This figure summarizes the principal findings of the study. In the MR analyses, TET2-CHIP showed the clearest associations with major cerebrovascular outcomes, including ischemic stroke and intracerebral hemorrhage, with hypertension as an additional associated phenotype. Other CHIP subtypes showed distinct patterns across cardiovascular outcomes, including atrial fibrillation and flutter, abdominal aortic aneurysm, and calcific aortic valve stenosis, highlighting marked subtype-specific heterogeneity. Cancer associations are also summarized to place these findings in a broader systemic context. The right panel presents the experimental validation strategy, in which *TET2* knockdown in THP-1-derived macrophages under oxLDL or LPS stimulation altered conditioned-medium effects on HUVECs and was associated with increased lipid accumulation, inflammatory activation, and endothelial dysfunction.

This Mendelian randomization analysis suggests that CHIP and its major subtypes exert heterogeneous effects across cerebrovascular outcomes, with additional associations observed for several cardiovascular conditions and cancer outcomes. These genetic findings were further supported by complementary cellular experiments focusing on TET2, which showed that *TET2* deficiency enhanced macrophage metabolic and inflammatory activation and aggravated endothelial dysfunction. Together, these findings support a role for CHIP as a subtype-specific contributor to disease risk within a broader cardiometabolic and inflammatory context (3, 6, 26). Rather than acting as a uniform exposure, CHIP exhibited bidirectional and gene-specific effects across disease outcomes, highlighting substantial biological heterogeneity among subtypes. The inclusion of cancer outcomes was intended to place these findings in a broader systemic context, given the growing links among CHIP, chronic inflammation, metabolic dysfunction and age-related multimorbidity (27). As summarized in Figure 5, the clearest subtype-specific signal was observed for TET2-CHIP in relation to cerebrovascular outcomes, whereas other CHIP subtypes showed distinct patterns across additional cardiovascular conditions and cancer outcomes.

Our analysis identified subtype-specific associations between CHIP and cerebrovascular outcomes, particularly IS and ICH, thereby extending prior epidemiological observations linking CHIP to stroke risk (13). Among the CHIP subtypes, TET2-CHIP showed the clearest and most coherent cerebrovascular signal, with increased risks of ischemic stroke and intracerebral hemorrhage together with an association with elevated blood pressure. This pattern suggests a distinct profile of cerebrovascular susceptibility related to TET2-CHIP. Consistent with these genetic observations, our cellular experiments showed that *TET2* knockdown enhanced macrophage inflammatory activation and lipid accumulation and promoted endothelial activation, providing biological support for the observed cerebrovascular associations. These findings are consistent with prior studies showing that *TET2*-deficient macrophages exhibit enhanced NLRP3 inflammasome-mediated IL-1β secretion and can promote vascular injury (28), while reduced TET2 activity in oxLDL-treated macrophages has also been linked to impaired autophagy and increased intracellular lipid accumulation (29).

In addition, ASXL1-CHIP showed a similar directional tendency for hemorrhagic and hypertensive phenotypes, DNMT3A-CHIP was positively associated with atrial fibrillation, and JAK2-CHIP showed inverse associations with ICH and atrial fibrillation, further underscoring marked subtype divergence. The associations with hypertension and atrial fibrillation further suggest that CHIP may influence cerebrovascular risk through related clinical phenotypes, which are established contributors to stroke risk and frequently cluster with cardiometabolic abnormalities in aging populations (30, 31). Beyond these stroke-related phenotypes, additional associations with AAA and calcific aortic valve stenosis indicate that subtype-specific CHIP effects also extend to broader cardiovascular conditions, consistent with emerging evidence linking clonal hematopoiesis to diverse cardiovascular disease manifestations (5, 32). Taken together, these findings suggest that subtype-specific CHIP effects are not restricted to direct cerebrovascular outcomes but also extend to related cardiovascular phenotypes that may shape stroke risk.

A defining feature of our results is the substantial heterogeneity across CHIP subtypes. Different driver mutations were associated with distinct and sometimes opposing patterns across cerebrovascular and related cardiovascular phenotypes. TET2-, DNMT3A-, and ASXL1-related CHIP generally aligned with higher-risk profiles, whereas JAK2-CHIP more often showed divergent or inverse associations. These contrasting patterns suggest that the consequences of clonal hematopoiesis depend on mutation-specific pathways rather than the mere presence of clonal expansion, as supported by experimental studies of CHIP subtypes (11, 15, 16, 28). This mutation-specific divergence may help explain inconsistencies across prior observational studies that treated CHIP as a single exposure.

These findings also place CHIP within a broader cardiometabolic and inflammatory context (33–36). Metabolic abnormalities such as dyslipidemia and chronic low-grade inflammation contribute to vascular dysfunction and cerebrovascular susceptibility, and our oxLDL experiments support a link between *TET2* deficiency and macrophage lipid accumulation under metabolic stress. Prior studies likewise support interactions between CHIP and metabolic-inflammatory processes, suggesting links with immune dysregulation and chronic metabolic stress (6, 7, 36). Together, these observations suggest that clonal hematopoiesis may interact with cardiometabolic risk factors through immune and inflammatory pathways rather than acting as an isolated hematologic phenomenon.

Cancer outcomes were included to place the cerebrovascular and cardiovascular findings in a broader systemic context. In this regard, metabolic-inflammatory processes may provide a shared biological background linking CHIP to both vascular and oncologic manifestations (5, 6, 19, 27). Several CHIP subtypes suggested parallel associations across cerebrovascular/cardiovascular and oncologic outcomes in our analysis. For example, DNMT3A-related CHIP showed concurrent associations with esophageal cancer and atrial fibrillation, whereas TET2-related CHIP was linked to both myeloid malignancy and cerebrovascular outcomes. These cross-disease patterns support the view that mutation-defined CHIP effects may extend across organ systems, although the main message of the present study remains the marked heterogeneity of subtype-specific cerebrovascular risk.

Overall, these findings highlight the relevance of CHIP to cerebrovascular risk within a broader cardiometabolic context and further emphasize the marked heterogeneity across CHIP subtypes. Among them, TET2-CHIP showed the clearest associations with cerebrovascular outcomes, supported by complementary experimental evidence linking TET2 with macrophage-driven inflammatory and metabolic alterations and endothelial dysfunction. These observations suggest that the vascular consequences of clonal hematopoiesis are not uniform, but instead depend on mutation-specific biological pathways that may shape neurovascular vulnerability in distinct ways. Future longitudinal studies integrating clonal dynamics, metabolic phenotyping and vascular risk profiling will be important not only to determine whether different CHIP subtypes confer distinct cerebrovascular risk trajectories or interact differently with cardiometabolic risk factors, but also to clarify the broader mutation-specific pathways underlying the heterogeneous clinical associations observed across CHIP subtypes beyond the TET2-related mechanisms supported by our current experimental data.

## Conclusion

This study provides genetic and experimental evidence that CHIP is associated with subtype-specific patterns of cerebrovascular risk within a broader cardiometabolic context. Among the major CHIP subtypes, TET2-CHIP showed the most consistent associations with cerebrovascular outcomes and was further supported by experimental findings linking TET2 deficiency to macrophage inflammatory and metabolic activation and to endothelial dysfunction. The marked heterogeneity observed across CHIP subtypes indicates that clonal hematopoiesis should not be considered a uniform exposure, but rather a mutation-defined condition with distinct clinical consequences. These findings support a mutation-informed framework for interpreting CHIP-related cerebrovascular susceptibility and further clarify how clonal hematopoiesis may interact with cardiometabolic processes relevant to stroke risk.

## Data Availability

The original contributions presented in the study are included in the article/Supplementary material, further inquiries can be directed to the corresponding authors.
