# Supplementary material for: Subtype-specific effects of clonal hematopoiesis on cerebrovascular and cardiometabolic disease risk
Source: Front Neurol. 2026 Apr 28;17:1830391. doi: 10.3389/fneur.2026.1830391 (PMC13160723; doi:10.3389/fneur.2026.1830391)

**Supplementary materials**

**Subtype-Specific Effects of Clonal Hematopoiesis on Cerebrovascular and Cardiometabolic Disease Risk**

**Figure S1**. Scatter plots of significant and nominal significant estimates from genetically predicted CHIP on (a) esophageal cancer, (b) liver cancer, (c) thyroid cancer, (d) myeloid leukemia, (e) non-melanoma skin cancer, (f) melanoma skin cancer and (g) bladder cancer.


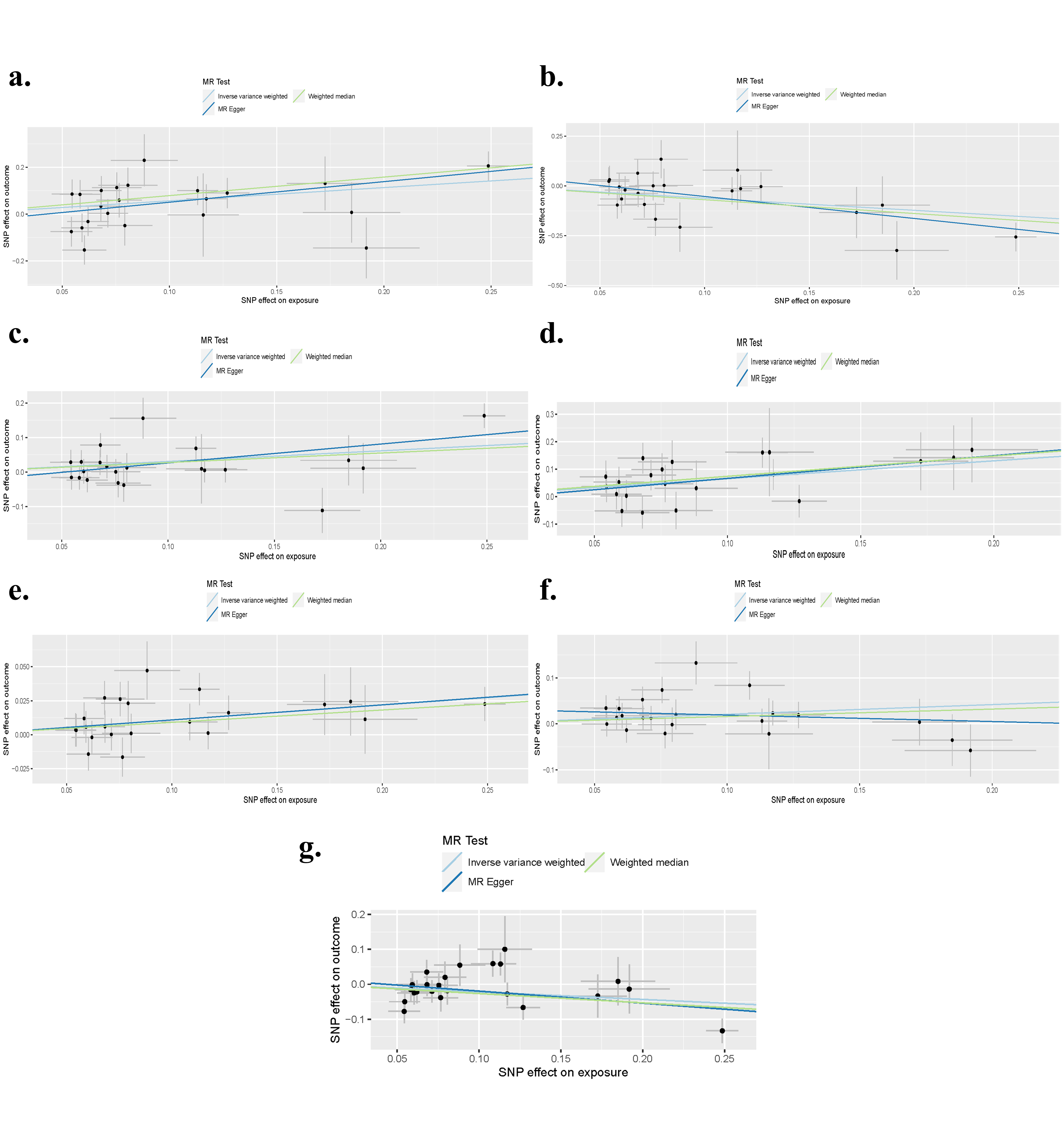


**Figure S2**. Scatter plots of significant and nominal significant estimates from genetically predicted DNMT3A-CHIP on (a) esophageal cancer, (b) liver cancer, (c) myeloid leukemia, (d) non-melanoma skin cancer, (e) breast cancer, (f) lymphoid leukemia and (g) kidney cancer.


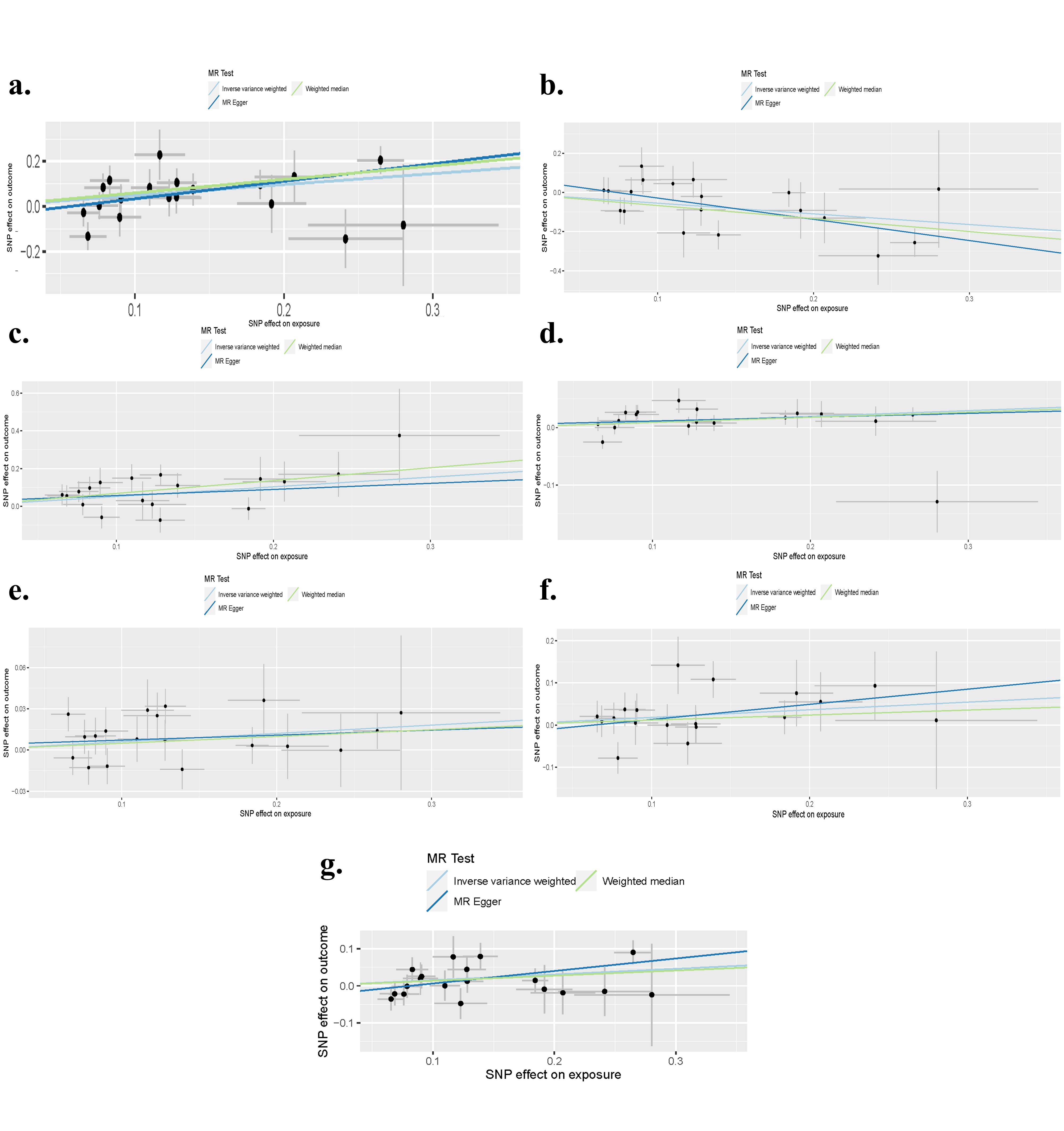


**Figure S3**. Funnel plots of significant and nominal significant estimates from genetically predicted CHIP on (a) esophageal cancer, (b) liver cancer, (c) thyroid cancer, (d) myeloid leukemia, (e) non-melanoma skin cancer, (f) melanoma skin cancer and (g) bladder cancer.


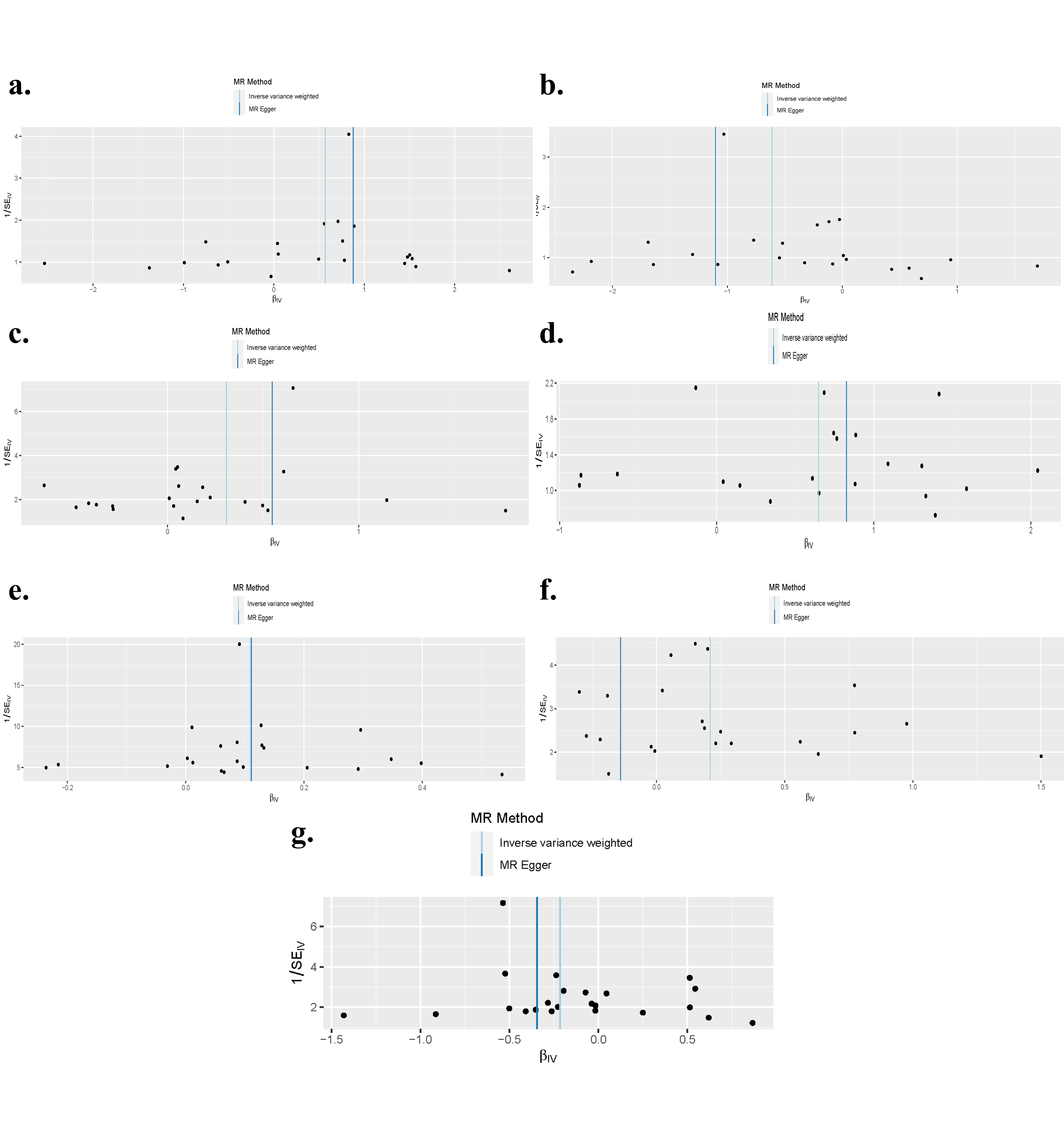


**Figure S4**. Funnel plots of significant and nominal significant estimates from genetically predicted DNMT3A-CHIP on (a) esophageal cancer, (b) liver cancer, (c) myeloid leukemia, (d) non-melanoma skin cancer, (e) breast cancer, (f) lymphoid leukemia and (g) kidney cancer.
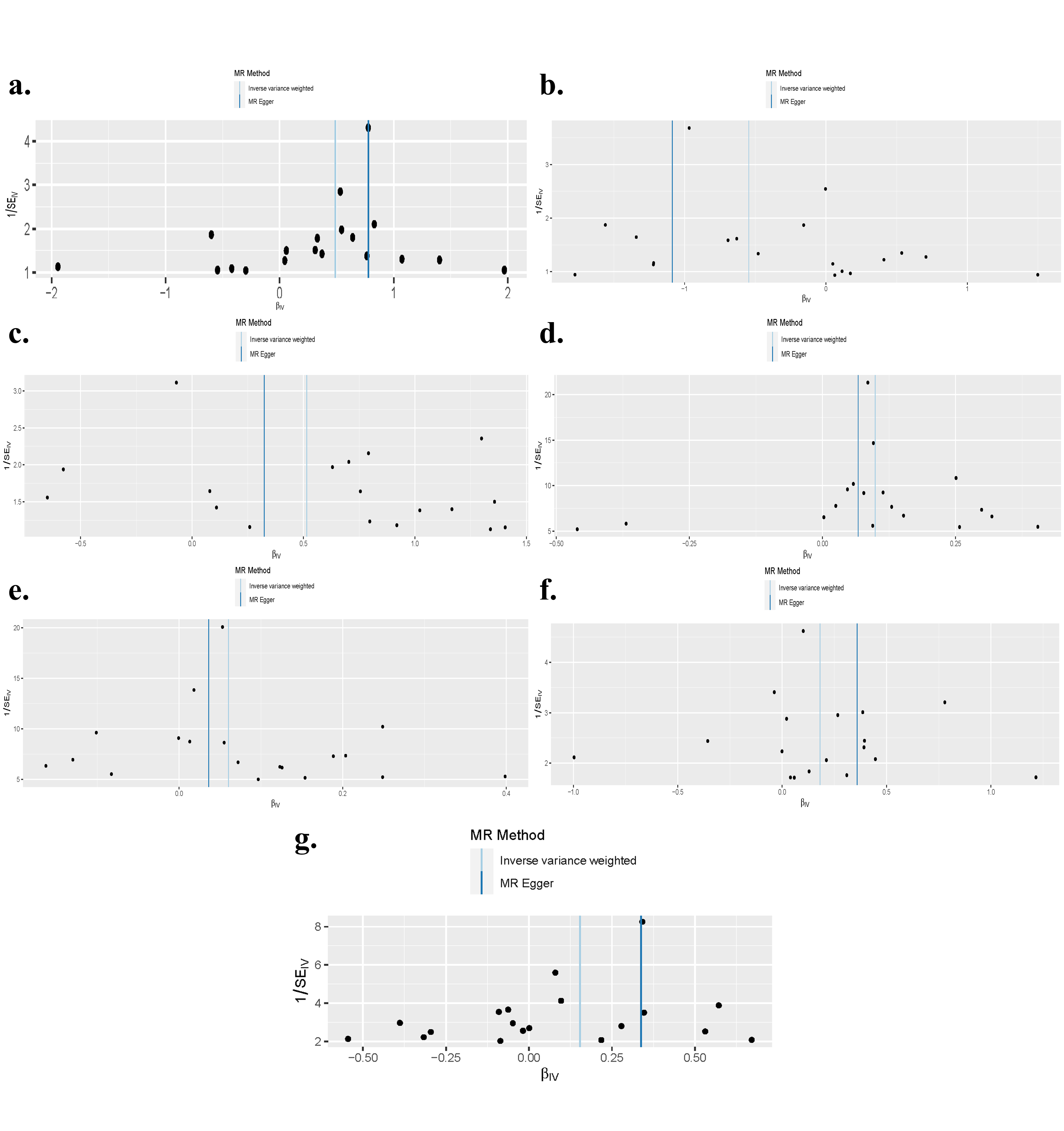


**Figure S5**. Leave-one-out plots of significant and nominal significant estimates from genetically predicted CHIP on (a) esophageal cancer, (b) liver cancer, (c) thyroid cancer, (d) myeloid leukemia, (e) non-melanoma skin cancer, (f) melanoma skin cancer and (g) bladder cancer.


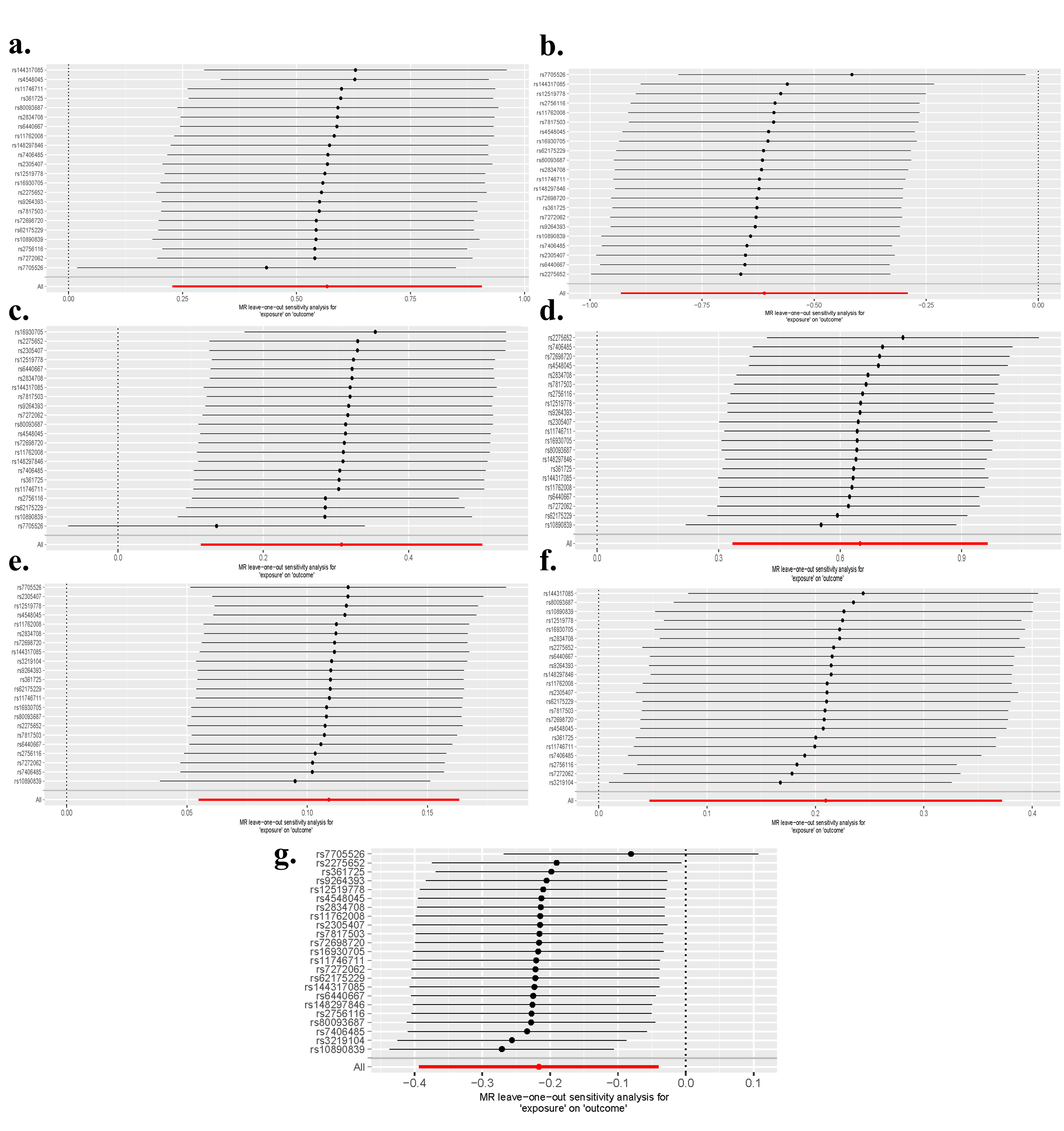


**Figure S6**. Leave-one-out plots of significant and nominal significant estimates from genetically predicted DNMT3A-CHIP on (a) esophageal cancer, (b) liver cancer, (c) myeloid leukemia, (d) non-melanoma skin cancer, (e) breast cancer, (f) lymphoid leukemia and (g) kidney cancer.


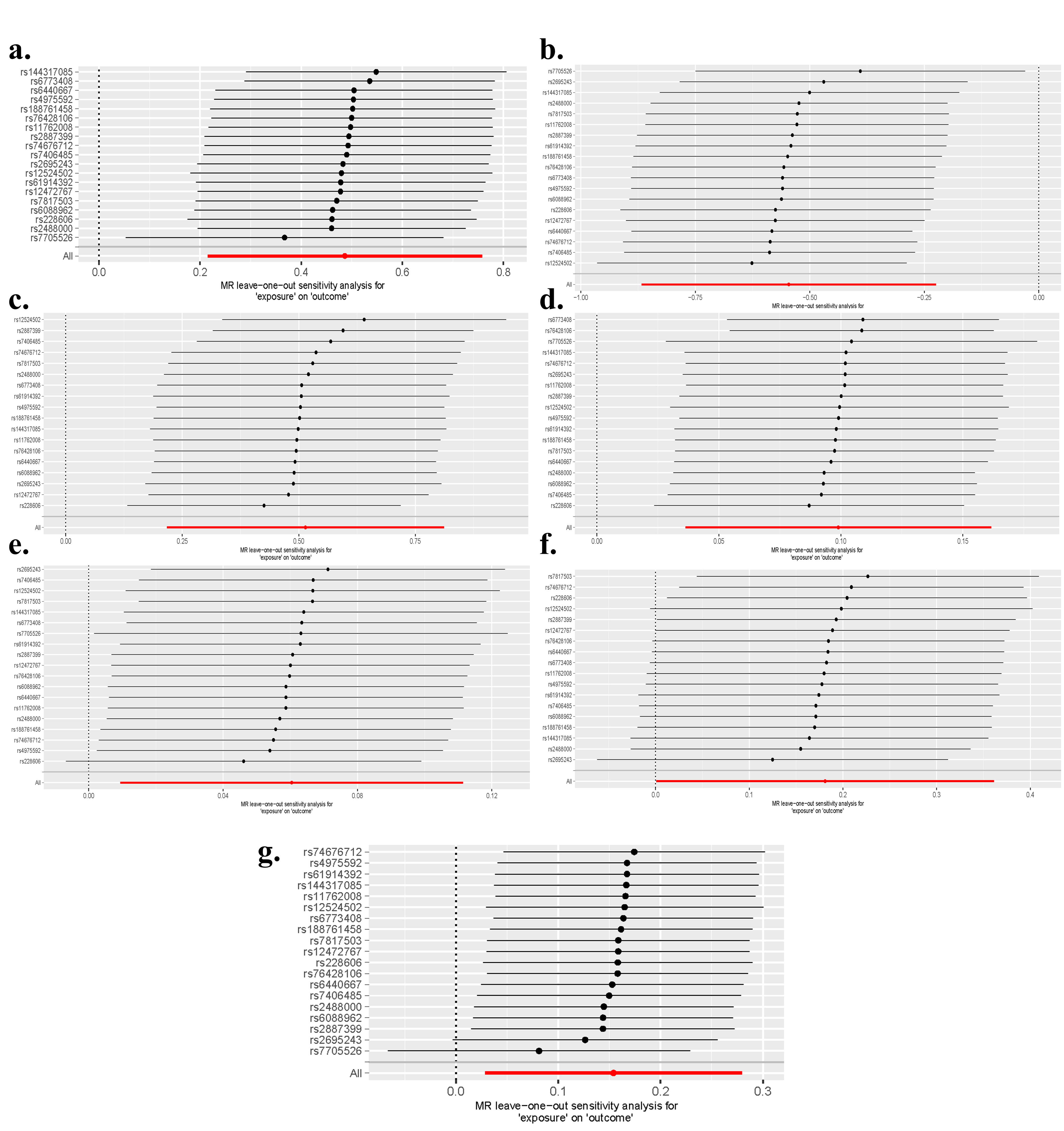


**Figure S7**. Scatter plots of significant and nominal significant estimates from genetically predicted CHIP on (a) abdominal aortic aneurysm, (b) atrial fibrillation and flutter, (c) intracerebral hemorrhage and (d) peripheral vascular disease.

**
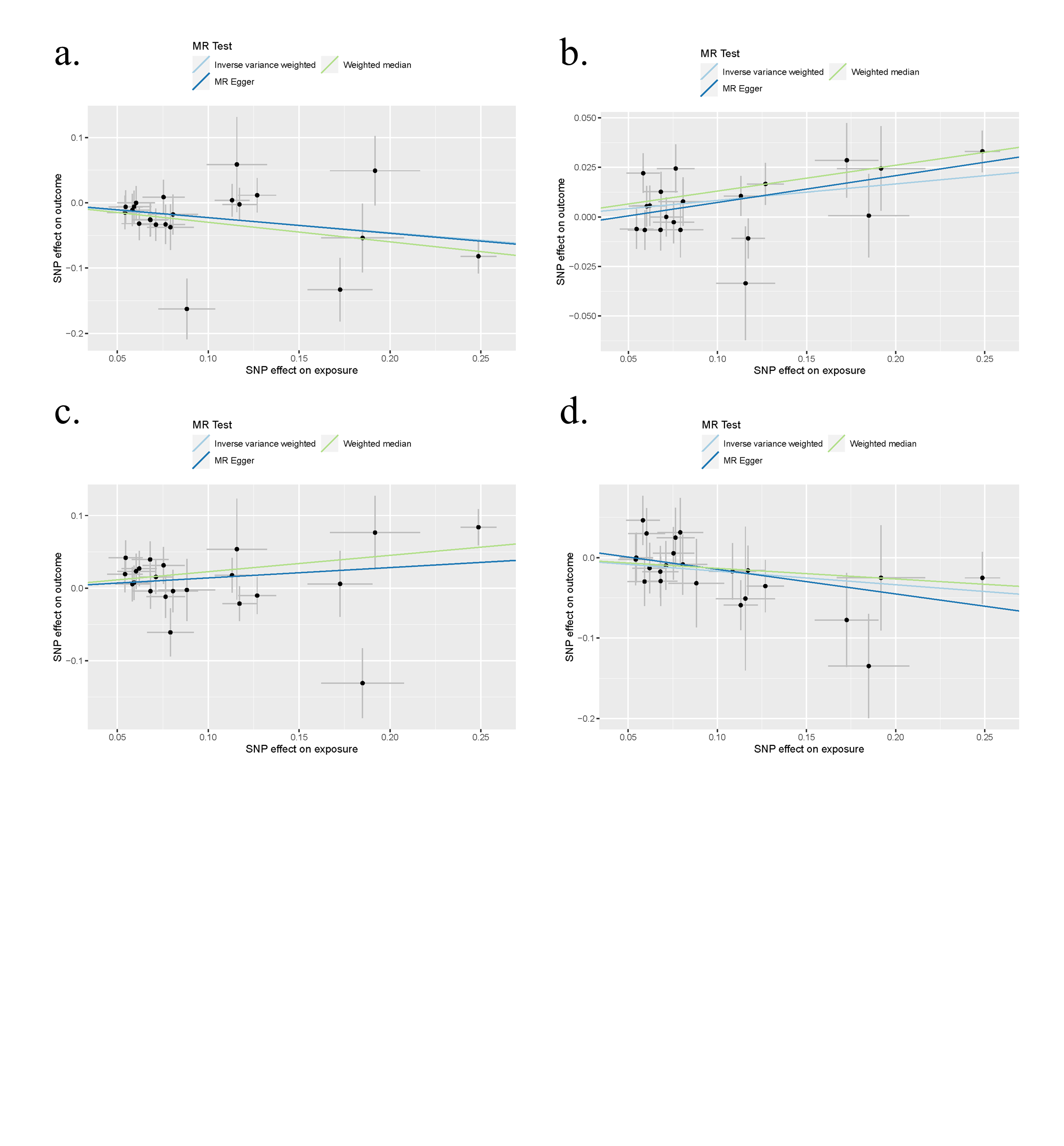
**

**Figure S8**. Scatter plots of significant and nominal significant estimates from genetically predicted DNMT3A-CHIP on (a) abdominal aortic aneurysm and (b) atrial fibrillation and flutter.

**
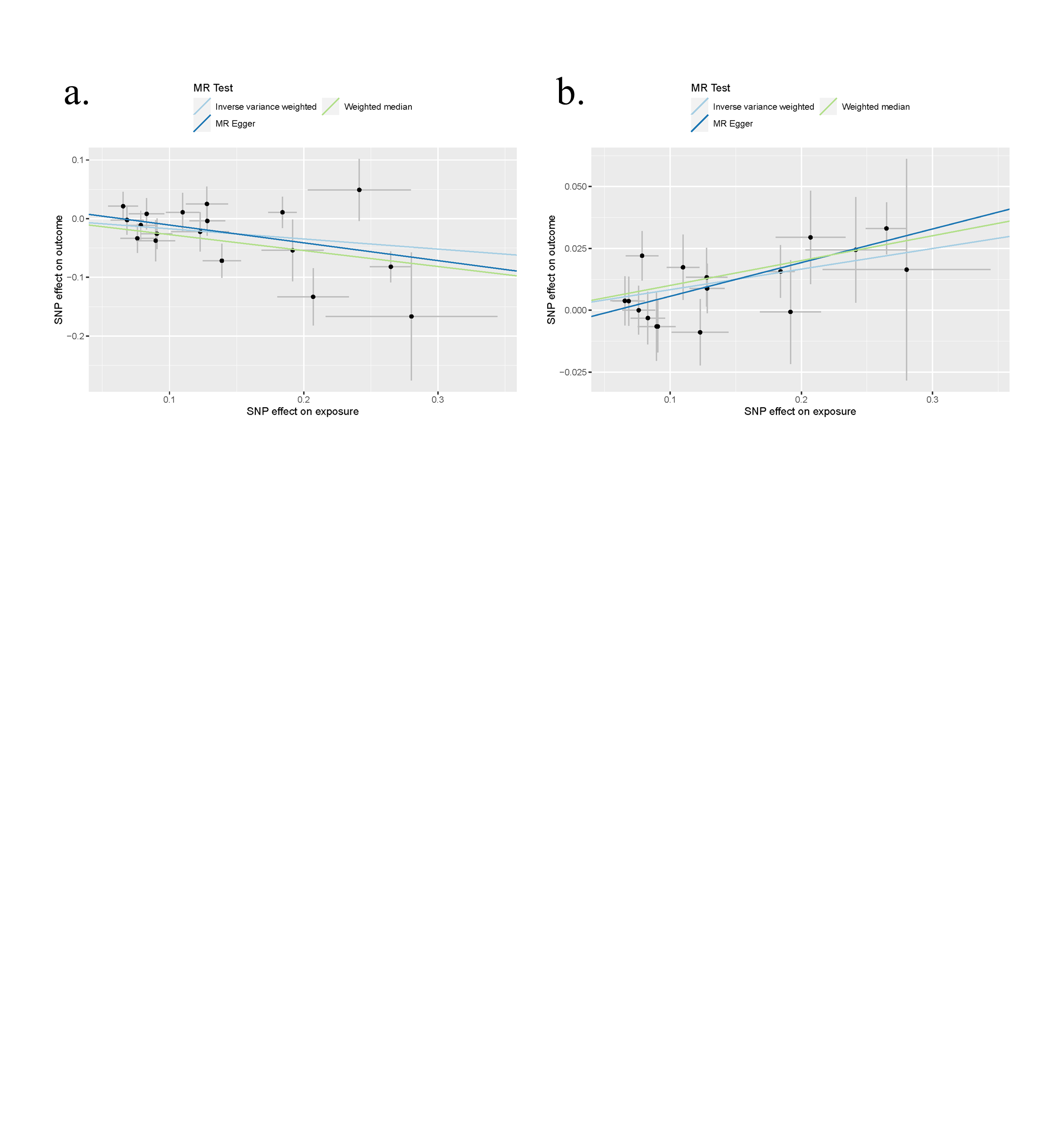
**

**Figure S9**. Funnel plots of significant and nominal significant estimates from genetically predicted CHIP on (a) abdominal aortic aneurysm, (b) atrial fibrillation and flutter, (c) intracerebral hemorrhage and (d) peripheral vascular disease.

**
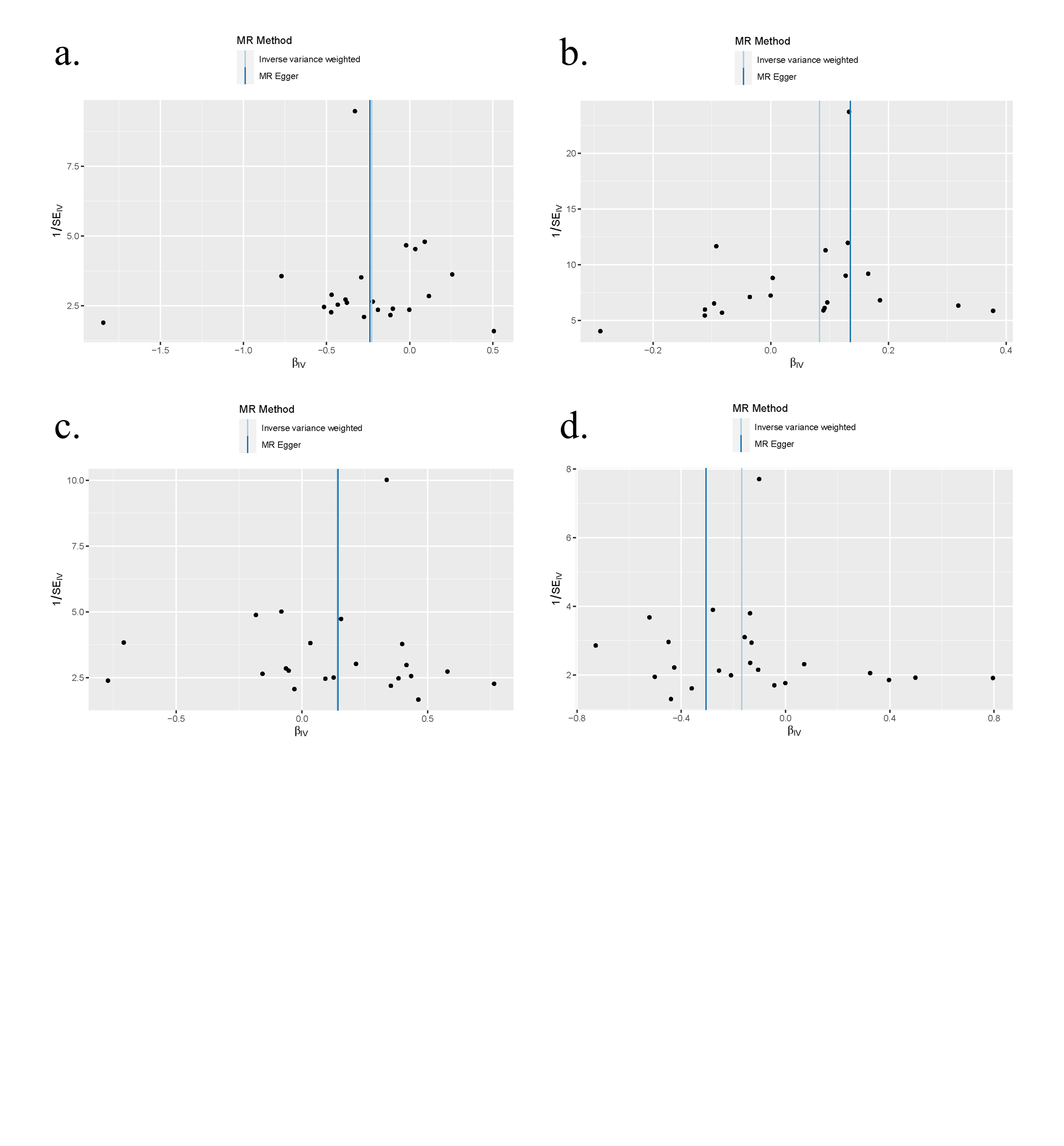
**

**Figure S10**. Funnel plots of significant and nominal significant estimates from genetically predicted DNMT3A-CHIP on (a) abdominal aortic aneurysm and (b) atrial fibrillation and flutter.

**
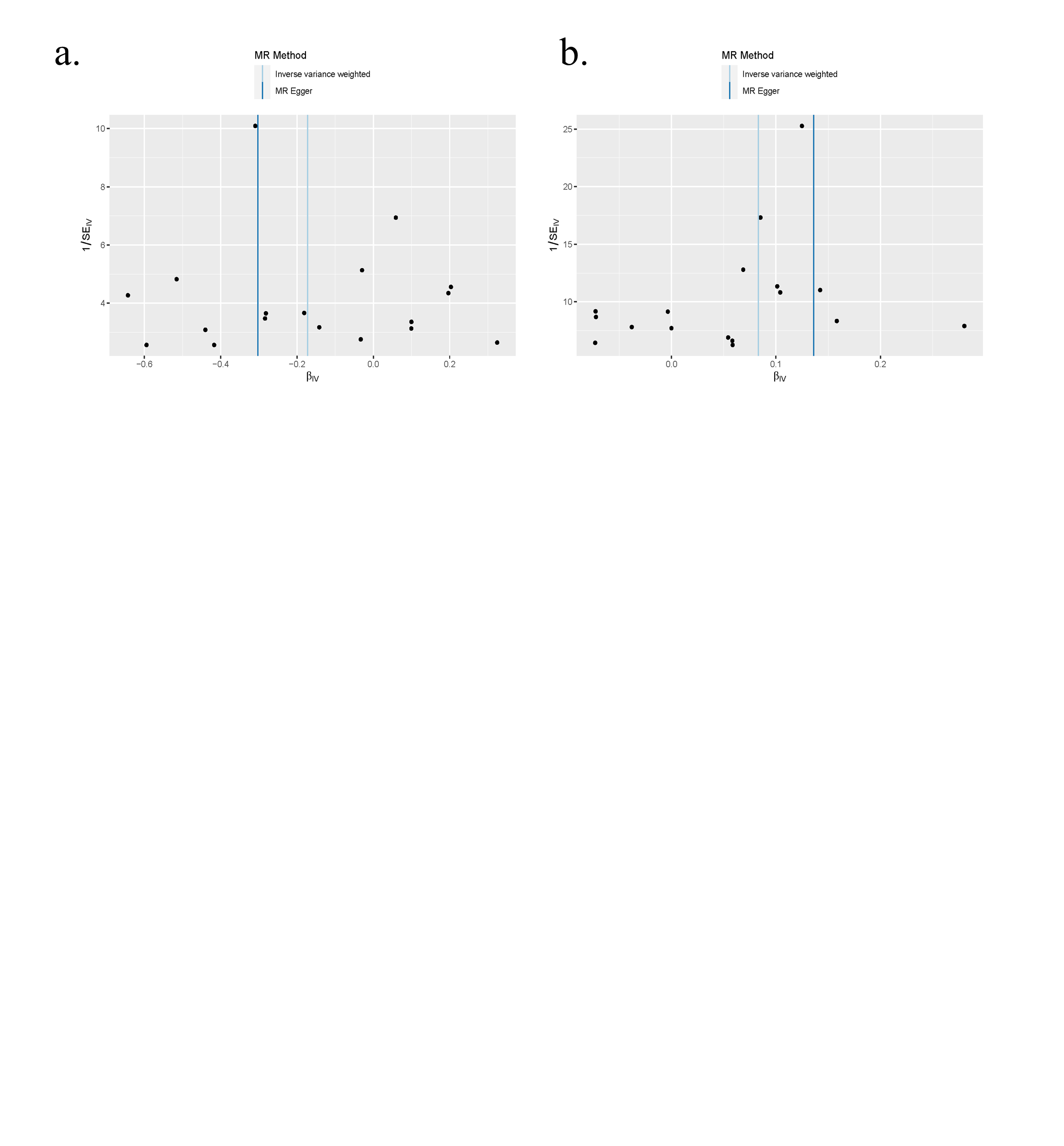
**

**Figure S11**. Leave-one-out plots of significant and nominal significant estimates from genetically predicted CHIP on (a) abdominal aortic aneurysm, (b) atrial fibrillation and flutter, (c) intracerebral hemorrhage and (d) peripheral vascular disease.

**
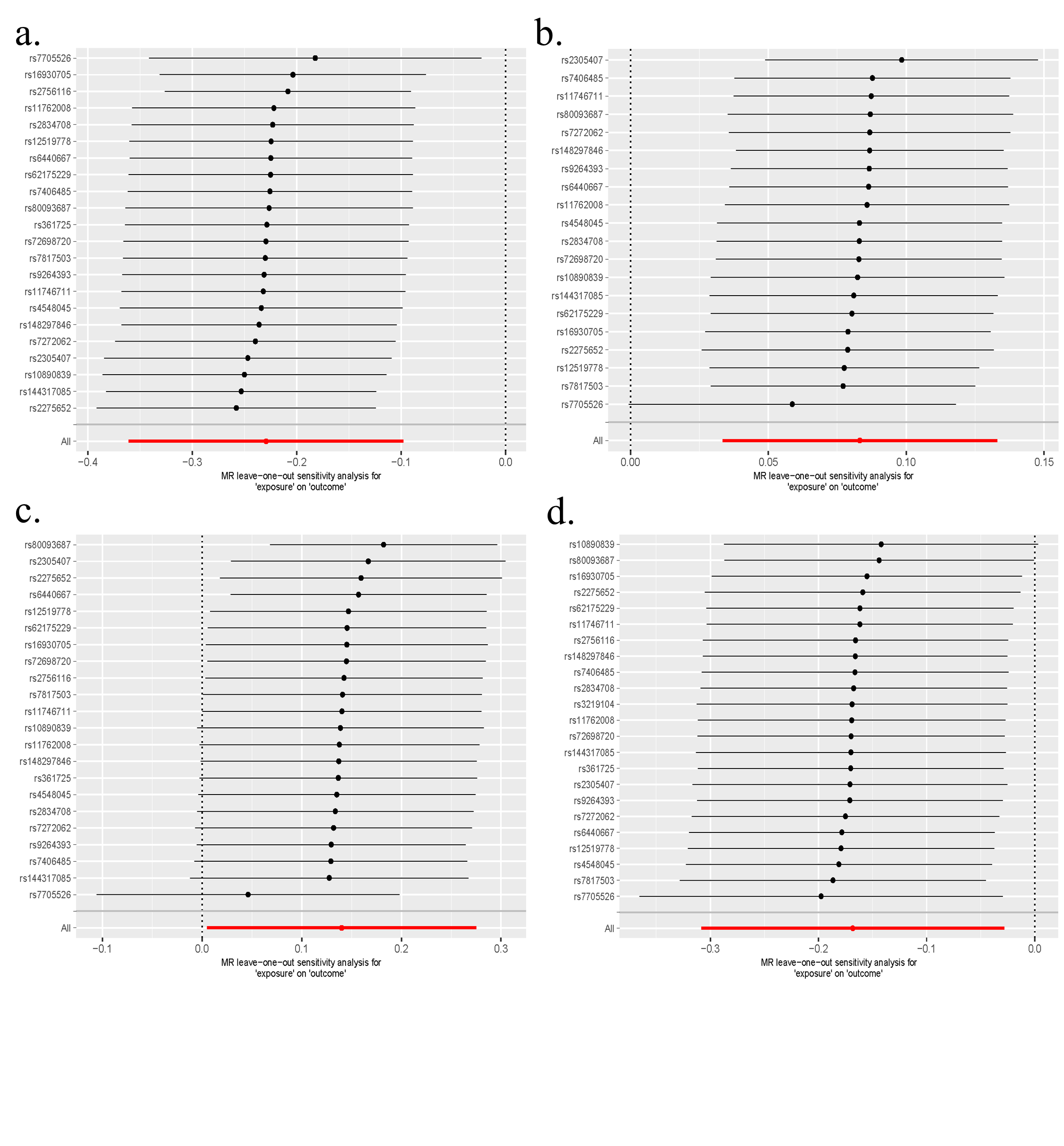
**

**Figure S12**. Leave-one-out plots of significant and nominal significant estimates from genetically predicted DNMT3A-CHIP on (a) abdominal aortic aneurysm and (b) atrial fibrillation and flutter.


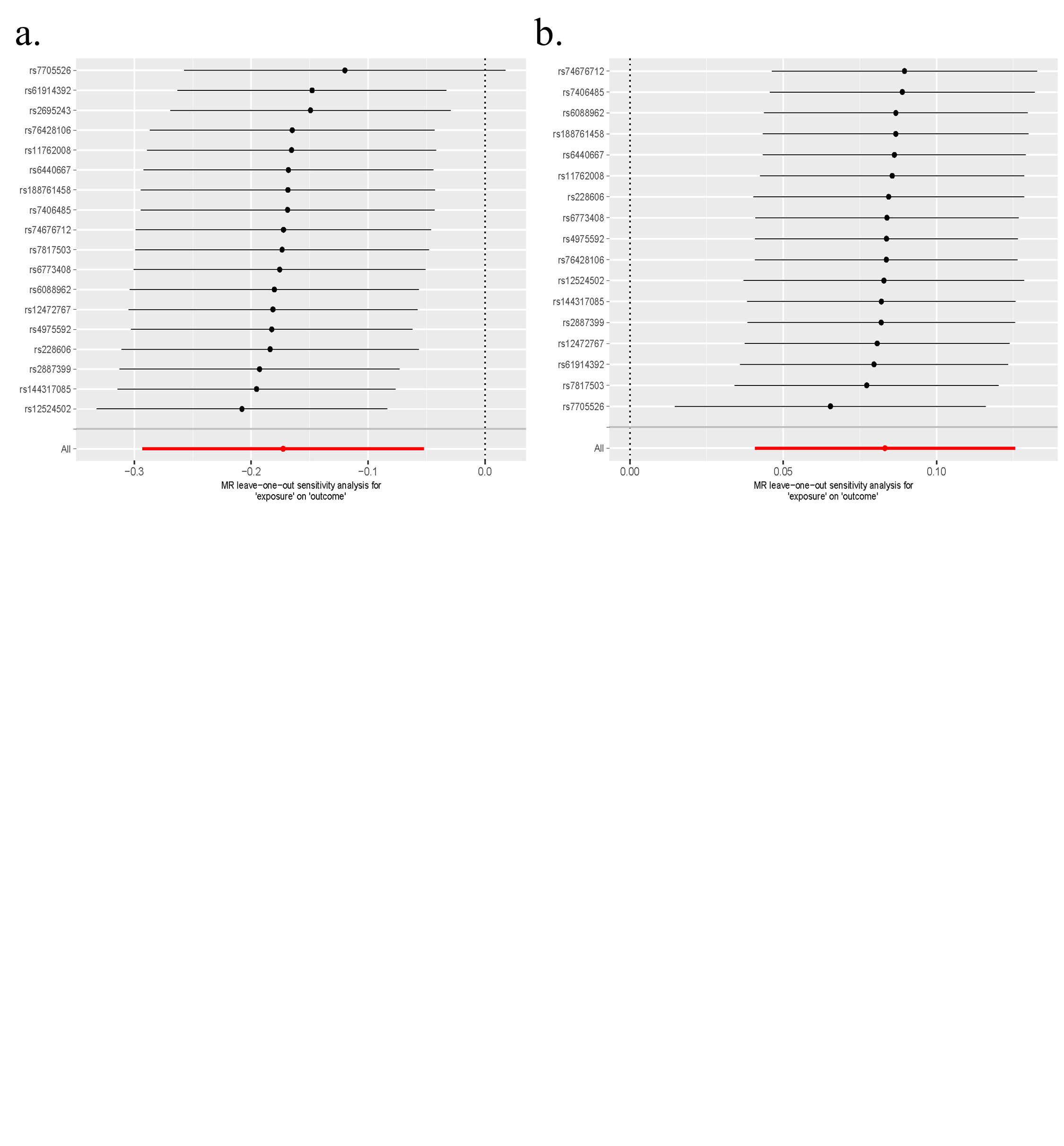

Supplement: Supplementary file 1 [file Supplementary_file_1.doc]
